# Phytotoxic and Antifungal Metabolites from *Curvularia crepinii* QTYC-1 Isolated from the Gut of *Pantala flavescens*

**DOI:** 10.3390/molecules23040951

**Published:** 2018-04-19

**Authors:** Caiping Yin, Liping Jin, Feifei Sun, Xiao Xu, Mingwei Shao, Yinglao Zhang

**Affiliations:** 1 School of Life Sciences, Anhui Agricultural University, Hefei 230036, China; yinglaozhang@aliyun.com; 2 College of Chemistry and Life Sciences, Zhejiang Normal University, Jinhua 321004, China; goldlp900906@163.com (L.J.); desunfeifei@163.com (F.S.); xuxiaoxiao0602@163.com (X.X.); jianting880720@126.com (M.S.)

**Keywords:** *Curvularia crepinii*, *Pantala flavescens*, phytotoxic activity, antifungal activity, macrolide, natural product

## Abstract

Four metabolites (**1**–**4**), including a new macrolide, *O*-demethylated-zeaenol (**2**), and three known compounds, zeaenol (**1**), adenosine (**3**), and ergosta-5,7,22-trien-3b-ol (**4**) were isolated and purified from *Curvularia crepinii* QTYC-1, a fungus residing in the gut of *Pantala flavescens*. The structures of isolated compounds were identified on the basis of extensive spectroscopic analysis and by comparison of the corresponding data with those reported in the literature previously. The new compound **2** showed good phytotoxic activity against *Echinochloa crusgalli* with an IC_50_ value of less than 5 µg/mL, which was comparable to that of positive 2,4-dichlorophenoxyacetic acid (2,4-D). Compound **1** exhibited moderate herbicidal activity against *E. crusgalli* with an IC_50_ value of 28.8 μg/mL. Furthermore, the new metabolite **2** was found to possess moderate antifungal activity against *Valsa mali* at the concentration of 100 µg/mL, with the inhibition rate of 50%. These results suggest that the new macrolide **2** and the known compound **1** have potential to be used as biocontrol agents in agriculture.

## 1. Introduction

Both weeds and plant fungal diseases have always been thought as big agricultural and environmental problems [1,2,3]. Organic synthetic herbicides and fungicides are widely used to control these issues. However, these synthetic chemicals may create serious problems to human health and lead to environmental pollution due to their slow biodegradation. Moreover, concerns of toxicity makes it necessary to discover more effective and safer herbicides and fungicides. The study of microbial secondary metabolites offers useful evidence in the investigation of new types of natural herbicides and fungicides that could be less harmful and more specific than the synthetic chemical agents currently used in agriculture [4,5,6]. Therefore, microbial natural products have attracted attention as reliable alternatives to synthetic agricultural chemicals [7].

Insect gut microorganisms constitute a rich and complex microbial population, and have gained attention as a resource of new bioactive metabolites [8,9,10]. In the proceeding of our ongoing efforts to find new natural herbicides and fungicides from insect gut microbes [11], we found that the extract from the solid fermentation of the fungal strain *Curvularia crepinii* QTYC-1, isolated from the gut of *Pantala flavescens*, exhibited potent phytotoxic activities against the radical growth of *Echinochloa crusgalli* and *Amaranthus retroflexus,* as well as antifungal activity against plant pathogens. Further examination of the crude extract led to the isolation of one new macrolide along with three known compounds. Here, we reported the details of the isolation, structure characterization, and bioactivities of these metabolites.

## 2. Results and Discussion

### 2.1. Identification of the Fungus

**Figure 1:**
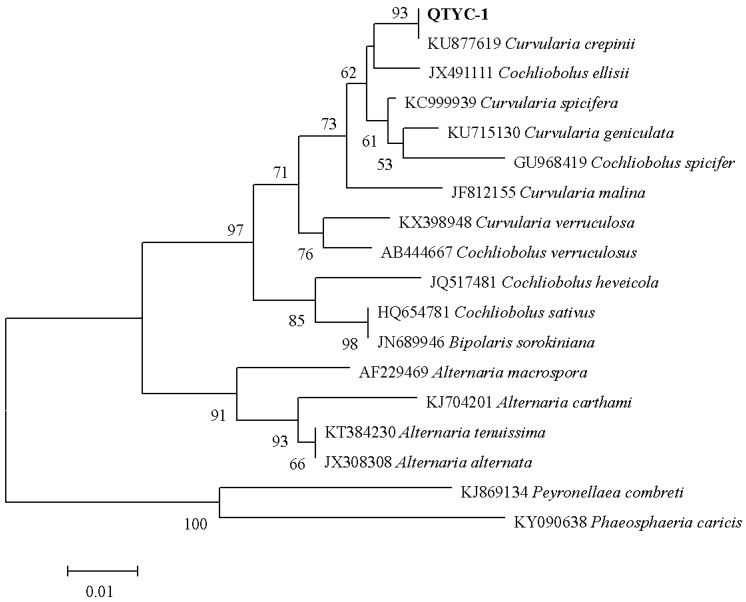
The phylogenetic tree of QTYC-1 based on the 5.8S rDNA sequences.

Colonies of QTYC-1 on malt-extract agar (MEA) grew quickly at 28 °C, covering the whole plate (9 cm in diameter) in 10 days. The morphological features of the strain QTYC-1 were similar to those of *Curvularia* sp [2], which produced four-celled ellipsoidal conidia. Phylogenetic taxonomy with the sequence alignment of ITS (Internal Transcribed Spacer) rDNA of the fungus was achieved with MEGA 5.0 software (http://www.megasoftware.net). The phylogenetic tree (Figure 1) indicated that the title fungus was closely related to *Curvularia crepinii* (KU877619), with the ITS sequence similarity of 100%. Combined with the morphological characteristics, the fungus was identified as *C. crepinii*.

### 2.2. Identification of Active Compounds

**Figure 2:**
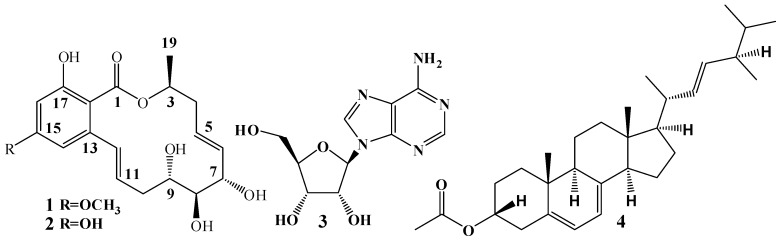
Chemical structures of secondary metabolites **1**–**4** of *C. crepinii* QTYC-1.

Bioassay-guided fractionation of the constituents in the ethyl acetate extract of *C. crepinii* QTYC-1 yielded a novel macrolide derivate **2** and three known active metabolites (Figure 2). 

Compound **2** was obtained as a white powder. Its molecular formula C_18_H_22_O_7_ was deduced from ESI-MS *m*/*z* 373 [M + Na]^+^, 701 [2M + H]^+^, which was consistent with the ^1^H-NMR and ^13^C-NMR data (Supplementary Materials, Figures S1–S6). The ^1^H-NMR (Table 1) of **2** indicated the presence of 1,3-disubstituted benzene ring (δ_H_ 6.45, *J* = 2.4 Hz; δ_H_ 6.27, *J* = 2.4 Hz), one methyl (δ_H_ 1.47), and two methylenes. Five proton signals at δ_H_ 3.93, 3.96, 3.80, 9.37, and 11.89 were assigned to hydroxy groups because no HMQC correlation was observed. The ^1^H- and ^13^C-NMR data were similar to those of zeaenol (**1**), except the *O*-demethylation at position 15 in **1** appeared to be OCH_3_ [12], which corresponded to the decrease in molecular weight of **2** by 14 amu compared to **1**. This was further confirmed by the HMBC correlation of HO-15 to C-14 (δ_C_ 108.6), C-15 (δ_C_ 163.4), C-16 (δ_C_ 102.6). Further confirmation was achieved by the HMBC correlation of H-14 to C-12 (δ_C_ 133.5), C-15, C-16, C-18 (δ_C_ 103.8); H-16 to C-14, C-18; H-19 to C-3 (δ_C_ 72.7), C-4 (δ_C_ 38.0); H-3 to C-1 (δ_C_ 172.4), C-5 (δ_C_ 127.9); H-4a to C-6 (δ_C_ 133.2), C-19 (δ_C_ 19.5); H-5 to C-3, C-7 (δ_C_ 73.7); H-6 to C-4; H-7 to C-5, C-8 (δ_C_ 78.9); H-10b to C-8, C-12; H-11 to C-9 (δ_C_ 74.2), C-13 (δ_C_ 144.8); H-12 to C-10 (δ_C_ 37.1), C-13, C-14, C-18; HO-17 to C-16, C-18. Thus, the structure of **2** was an *O*-demethylated derivative of **1** and was determined as (3S*,5*E*,7S*,8S*,9S*,11*E*)-7,8,9,15,17-pentahydroxy-3-methyl-3,4,7,8,9,10-hexahydro-1*H*-benzo[c][1]oxacyclotetradecin-1-one. The planar structure of compound **2** has been described previously as an intermediate in the synthesis of derivative **1** [13]. However, it had not been described from nature and the relative configuration of C-8 in **2** has not been previously assigned. 

The other secondary metabolites were identified as zeaenol (**1**) [12], adenosine (**3**) [14], and ergosta-5,7,22-trien-3b-ol (**4**) [15], by comparing their MS and NMR spectroscopic data with those reported in the literatures.

The genus of *Curvularia* species was well-known to produce a variety of secondary metabolites. Previous investigations led to the isolation of several compounds such as antimicrobial curvulone A–B and curvularides A–E [16,17], motility inhibitor murranofuran A [18], cytotoxic pentanorlanostane, and pyrenocine J [19,20]. However, to the best of our knowledge, this is the first report of the new macrolide **2** isolated from the title strain *C. crepinii* QTYC-1, a fungus residing in the *P. flavescens* gut. 

### 2.3. Phytotoxic Activity of the Bioactive Metabolites

**Figure 3:**
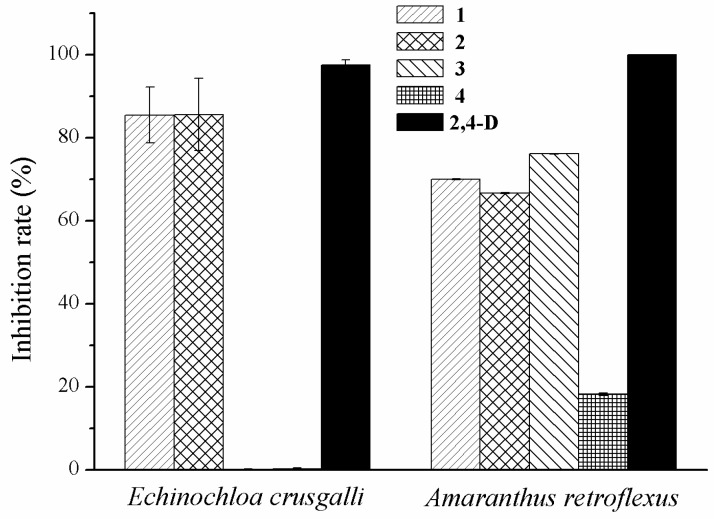
The phytotoxic effects of compounds **1**–**4** on radicle growth of *E. crusgalli* and *A. retroflexus*.

Compounds **1**–**4** were assayed for their ability to inhibit radicle growth of *E. crusgalli* and *A. retroflexus* using a Petri dish bioassay. The result (Figure 3) showed that compounds **1** and **2** were very active in reducing radicle growth of *E. crusgalli* with inhibition rates of 85.5% and 85.6%, respectively, which was comparable to the activity of positive 2,4-dichlorophenoxyacetic acid (2,4-D) with an inhibition rate of 97.5% under the concentration of 100 µg/mL. Compounds **1**–**3** showed potent phytotoxic activity against *A. retroflexus* with inhibition rates of 66~76% at the concentration of 100 μg/mL. However, compound **4** showed weak inhibitory effect against *A. retroflexus* and no obvious inhibitory effects were displayed by compounds **3** and **4** against *E. crusgalli* in this bioassay.

**Figure 4:**
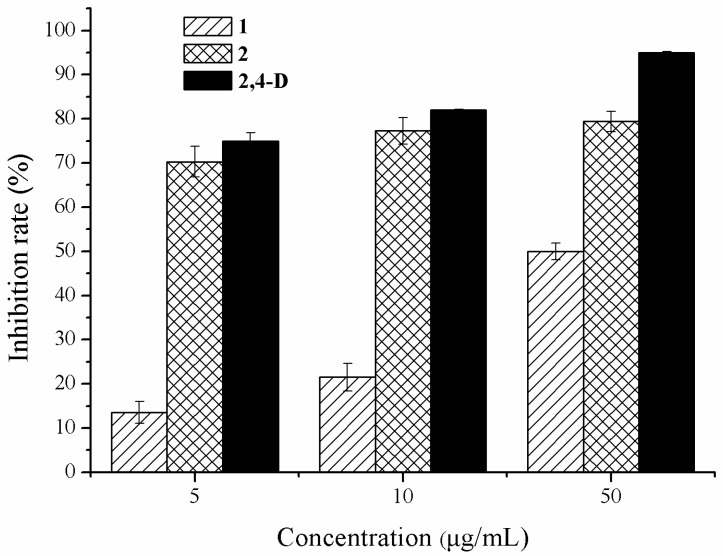
The phytotoxic effects of compounds **1** and **2** with different concentrations on radicle growth of *E. crusgalli*.

Compounds **1** and **2** were further tested to elucidate their phytotoxic activity at different concentrations against *E. crusgalli*, compared to 2,4-D co-assayed as a positive reference. The result (Figure 4) showed that compounds **1** and **2** exhibited phytotoxic activity in a dose-dependent manner (5–50 µg/mL). Furthermore, the new compound **2** exhibited higher phytotoxic activity than compound **1** at the same concentration. The 50% inhibitory concentration (IC_50_ value) of the new compound **2** was less than 5 µg/mL, which was comparable to that of positive 2,4-D. Compound **1** exhibited moderate phytotoxic activity against *E. crusgalli* with an IC_50_ value of 28.8 μg/mL.

### 2.4. In Vitro Effect on Mycelial Growth of Phytopathogenic Fungi

The effects of metabolites **1**–**4** against the mycelial growth of five phytopathogenic fungi were evaluated in vitro under the concentration of 100 µg/mL (Table 2). The results showed that metabolite **2** possessed moderate antifungal activity against *Valsa mali* with an inhibition rate of 50% when compared with that of positive cycloheximide. However, it weakly inhibited the growth of *Gibberella sanbinetti*, *Alternaria solani,* and *Fusarium oxysporum* f.sp. *mornordicae* with an inhibition rate of less than 45%. In general, compounds **1**, **3** and **4** exhibited weak activities against all tested phytopathogenic fungi with inhibition rates of less than 42%.

In summary, we identified one new macrolide, together with three known compounds, from *C. crepinii* QTYC-1, a fungus residing in the gut of *Pantala flavescens*. The new macrolide **2** and the known compound **1** attenuated the radicle growth of *E. crusgalli* and *A. retroflexus*, and **2** possessed moderate antifungal activity against *V. mali* in vitro. These results suggested that the compounds **1** and **2** have some potential as agents for weeds or pathogenic fungal control. Further studies will be carried out to better understand the mechanism of action associated with phytotoxic and antifungal effects. In addition, the discovery of our study provided additional evidence that the special microorganisms in uninvestigated habitats, just like the title strain, may inspire the discovery of chemical agents with interesting biological activity. 

## 3. Materials and Methods

### 3.1. Isolation and Identification of Strain QTYC-1

The strain was isolated based on methods described previously [21]. *P. flavescens* were collected from the suburb of Jinhua, Zhejiang Province, PR China during the growing season. The insects were brought to the lab and starved for 24 h. The insects were surface-sterilized in 75% ethanol for 3 min, followed by rinsing in sterilized water three times (30 s each). They were then degutted using sterile forceps. The guts were lightly homogenized, and dilution series (10^−1^, 10^−2^, 10^−3^) were spread-plated on malt-extract agar (MEA) medium (consisting of 20 g malt extract, 1 g peptone, 20 g sucrose, 20 g agar in 1 L of distilled water) containing antibacterial antibiotics (chloramphenicol and penicillin, 100 µg/mL each) to isolate the fungal strain. All of the plates were incubated aerobically in a chamber for 72 h at 28 ± 0.5 °C and any fungal colonies that formed were sub-cultured on new MEA medium to obtain pure cultures. The isolated strain was preserved on MEA slants at 4 °C until use. The title strain was deposited at the China Center for Type Culture Collection (CCTCC) as CCTCC M 2014308, and identified by comparing the morphological character and ITS sequence to those of standard records.

### 3.2. Microbial Fermentation

Based on the methods detailed elsewhere with trivial changes [22], the fungal strain was cultured on MEA medium at 28 ± 0.5 °C for four days. Then, pieces of fresh mycelium were inoculated into 250-mL Erlenmeyer flasks, each containing 100 mL of malt-extract (ME) liquid medium. After three days of incubation at 28 ± 0.5 °C on rotary shakers at 170 rpm, 10 mL cultural liquid was transferred as a seed into 1-L Erlenmeyer flasks, each containing 160 g of rice and 200 mL distilled H_2_O, and incubated at 28 ± 0.5 °C for 40 days. 

### 3.3. Isolation and Characterization of Secondary Metabolites

The total solid fermentation product in 40 Erlenmeyer flasks was extracted with ethyl acetate (4 × 10 L) at room temperature. The solvent was then evaporated in vacuo to afford a crude extract (50.1 g). The extract was subjected to chromatography over a silica-gel column eluting with a stepwise gradient of CH_2_Cl_2_/MeOH (100:0–100:8, *v/v*) to give five fractions (Fr-1 to Fr-5). Fr-1 was further chromatographed over silica gel (CH_2_Cl_2_/MeOH, 100:0–100:2) to give four sub-fractions (R1–R4). Compound **4** (6.0 mg) was crystallized from the MeOH solution of sub-fraction R2. Fr-3 (CH_2_Cl_2_/MeOH, 100:2) was repeatedly purified on silica gel (CH_2_Cl_2_/MeOH, 100:1−100:2) to yield compounds **1** (100.2 mg) and **2** (7.6 mg). Fr-5 (CH_2_Cl_2_/MeOH, 100:8) was loaded onto a Sephadex LH-20 column (MeOH) to give compound **3** (5.0 mg). 

Structural identifications of the secondary metabolites were made according to the spectroscopic analysis. The electrospray ionization mass spectrometry (ESI-MS) spectra were collected on a Time-of-Flight Mass Spectrometer G6230AA (Agilent Technologies, Santa Clara, CA, USA). ^1^H nuclear magnetic resonance (NMR), ^13^C-NMR and distortionless enhancement by polarization transfer (DEPT) spectra were acquired on a Bruker AVANCE-600 (Bruker, Fällanden, Switzerland), and chemical shifts were obtained in δ (ppm) by referring to the tetramethylsilane (TMS) and solvent signals as internal standards. HMQC and HMBC experiments were optimized for 145.0 and 8.0 Hz, respectively.

### 3.4. Phytogrowth Inhibitory Bioassay of Metabolites

The phytotoxic effects of compounds **1**–**4** were evaluated on the radicle growth of *E. crusgalli* and *A. retroflexus* according to the Petri dish bioassay [21]. Briefly, seeds were firstly surface-sterilized with sodium hypochlorite (1%) and washed with sterile distilled water before germination. Different concentrations of compounds **1**–**4** were prepared with acetone. A 5.0-mL sample solution was added to 9-cm diameter Petri dishes on filter paper disks. To avoid the toxic effect of solvents, filter papers were placed in a cabinet to evaporate the solvent. Subsequently, 5.0 mL of distilled water was added to each Petri dish. Then, 30 pre-germinated seeds were placed in the Petri dishes. 2,4-dichlorophenoxy acetic acid (2,4-D) was used as the positive reference. Dishes were then kept in an incubator at 25 °C under dark conditions. After two days, root length was checked and compared with the untreated control. The inhibition percent was calculated using the formula as follows:

Inhibition (%) = (L_control_ − L_treatment_)/L_control_ × 100

where L_control_ = radicle length of the seedlings in the control and L_treatment_ = radicle length of the seedlings treated.

### 3.5. In Vitro Effect on Mycelial Growth of Phytopathogenic Fungi

Antifungal bioactivities against phytopathogenic fungi were carried out by the mycelium growth rate method as described previously with slight changes [23,24]. Purified metabolites were dissolved in an aqueous solution (composed of 4% Tween-80 and 1% DMSO). Solutions of purified chemicals were mixed with MEA in a Petri dish (9 cm in diameter). Cycloheximide was used as the positive control. After inoculation of the fungal mycelia onto the center of the solid medium, the dishes were incubated in the dark at 28 ± 0.5 °C. When the fungal mycelium reached the edges of the control dishes, the antifungal activities were calculated. The formula for counting the percentage of growth inhibition is as follows:

Inhibition (%) = (1 − Da/Db) × 100

where Da is the diameter of the growth zone in the experimental dish (mm) and Db is the diameter of the growth zone in the control dish (mm). 

### 3.6. Statistical Analysis 

All experiments were performed in triplicate, and data are shown as mean values ± standard deviation. A least significant difference (LSD) test with a confidence interval of 95% was used to compare the means.

## Figures and Tables

**Table 1:** ^1^H-NMR and ^13^C-NMR data of compound **2** in acetone-*d*_6_.

Position	δ_H_, Mult. (*J* in Hz)	δ_C_
1		172.4
2		
3	5.32, m	72.7
4a	2.54, m	38.0
4b		
5	5.99, m	127.9
6	5.70, dd (15.4, 7.3)	133.2
7	4.15, t ( 7.3, 7.6)	73.7
8	3.50, d (7.6)	78.9
9	3.78, m	74.2
10 a	2.33, s	37.1
10 b	2.45, m	
11	5.99, m	131.4
12	7.15, d (15.6)	133.5
13		144.8
14	6.45, d (2.4)	108.6
15		163.4
16	6.27, d (2.4)	102.6
17		166.3
18		103.8
19	1.47, d (6.2)	19.5
7-OH	3.93, d (2.3)	
8-OH	3.96, d (3.0)	
9-OH	3.80, s	
15-OH	9.37, s	
17-OH	11.89, s	

**Table 2:** Inhibition rate of the compounds of QTYC-1 against phytopathogenic fungi (in %) ^a^.

Phytopathogens	1	2	3	4	Cycloheximide ^b^
*V. mali*	30.4 ± 0.6	50.0 ± 0.6	NI	28.9 ± 0.3	100 ± 0.0
*D. gregaria*	NI	NI	NI	NI	99.7 ± 1.6
*G. sanbinetti*	29.5 ± 0.1	30.4 ± 0.1	28.4 ± 0.1	NI	99.0 ± 0.3
*A. solani*	20.1 ± 0.1	35.2 ± 0.1	4.4 ± 0.1	16.4 ± 0.1	98.2 ± 2.7
*F. oxysporum* f.sp. *mornordicae*	41.1 ± 0.1	42.2 ± 0.1	31.3 ± 0.0	7.5 ± 0.1	98.7 ± 0.6

^a^ The concentration of compounds was 100 µg/mL; results were presented as the mean ± standard deviation for triplicate experiments. ^b^ Cycloheximide was used as the positive control. NI = not inhibited.

## Supplementary Materials

Supplementary materials can be accessed online. Figure S1: ^1^H-NMR spectrum (600 MHz) of the compound **2** in Acetone-*d*_6_. Figure S2: ^13^C-NMR spectrum (150 MHz) of compound **2** in Acetone-*d*_6_. Figure S3: DEPT 135 spectrum of compound **2** in Acetone-*d*_6._ Figure S4: ^1^H-^1^H COSY spectrum of compound **2** in Acetone-*d*_6_. Figure S5: HMQC spectrum of compound **2** in Acetone-*d*_6_. Figure S6: HMBC spectrum of compound **2** in Acetone-*d*_6_.
