# Supplementary material for: Phytotoxic and Antifungal Metabolites from Curvularia crepinii QTYC-1 Isolated from the Gut of Pantala flavescens
Source: Molecules. 2018 Apr 19;23(4):951. doi: 10.3390/molecules23040951 (PMC6017354; doi:10.3390/molecules23040951)
Supplement: Supplementary file 1 [file molecules-23-00951-s001.pdf]

Supplementary Materials:

Phytotoxic and Antifungal Metabolites from *Curvularia crepinii* QTYC-1 Isolated from the Gut of *Pantala flavescens*

Caiping Yin, Liping Jin, Feifei Sun, Xiao Xu, Mingwei Shao, Ying-Lao Zhang

Figure S1. ^{1}H-NMR spectrum (600 MHz) of the compound **2** in Acetone-d_6.

Figure S2. ^{13}C-NMR spectrum (150 MHz) of compound **2** in Acetone-d_6.

Figure S3. DEPT 135 spectrum of compound **2** in Acetone-d_6.

Figure S4. ^{1}H-^{1}H COSY spectrum of compound **2** in Acetone-d_6.

Figure S5. HMQC spectrum of compound **2** in Acetone-d_6.

Figure S6. HMBC spectrum of compound **2** in Acetone-d_6.

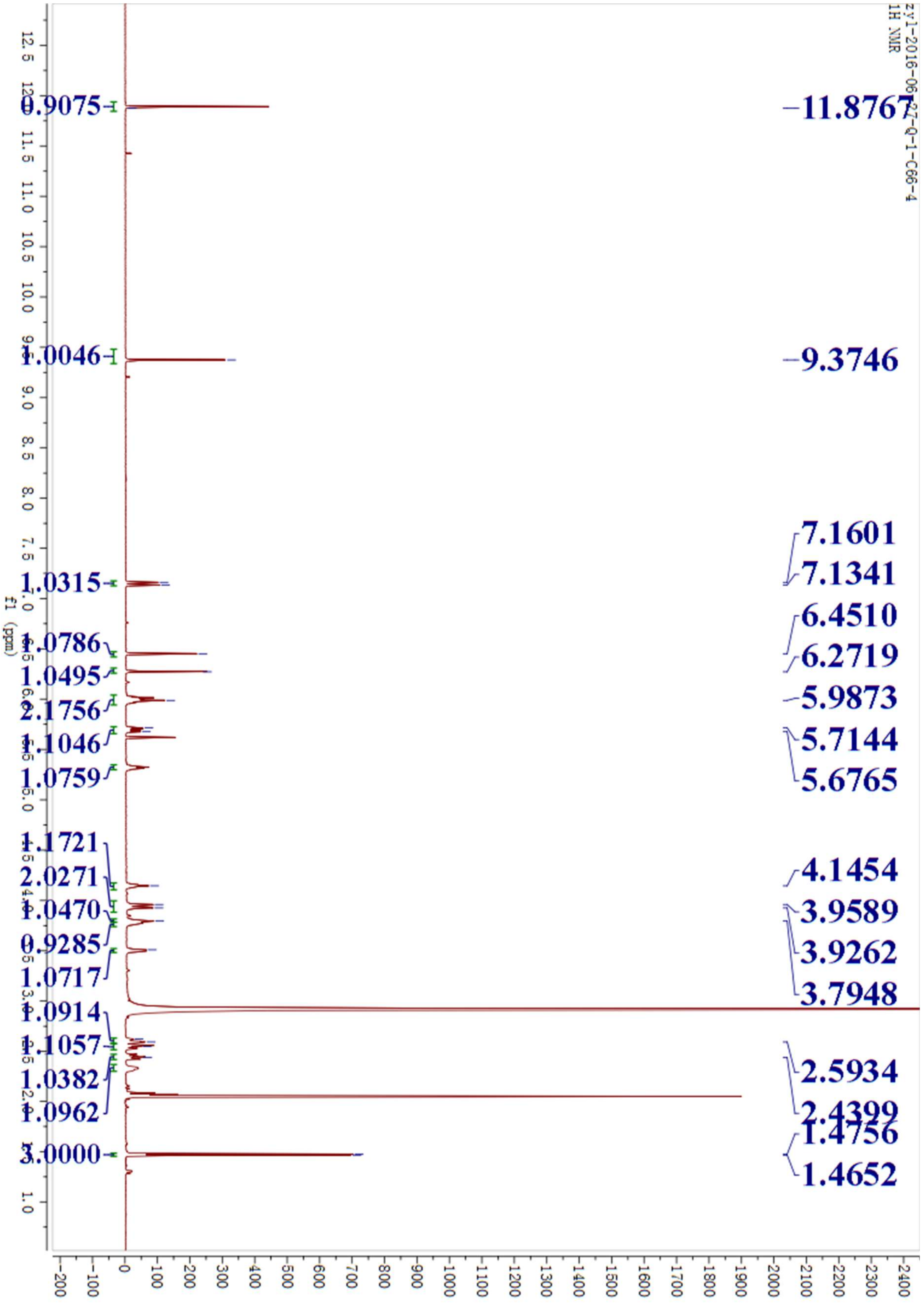

Figure S1. ^{1}H-NMR spectrum (600 MHz) of the compound **2** in Acetone-d_6.

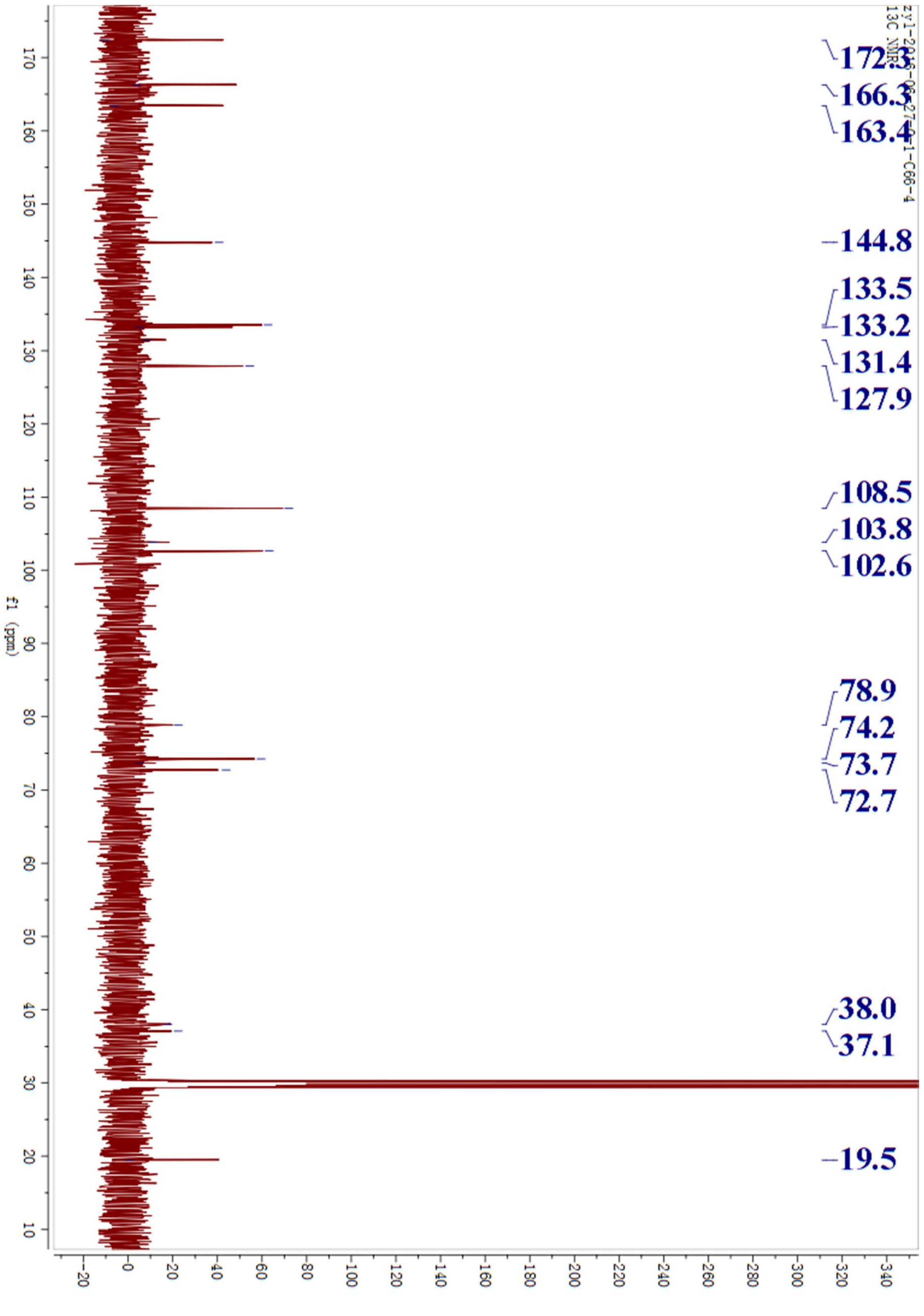

Figure S2. ^{13}C-NMR spectrum (150 MHz) of compound **2** in Acetone-d_6.

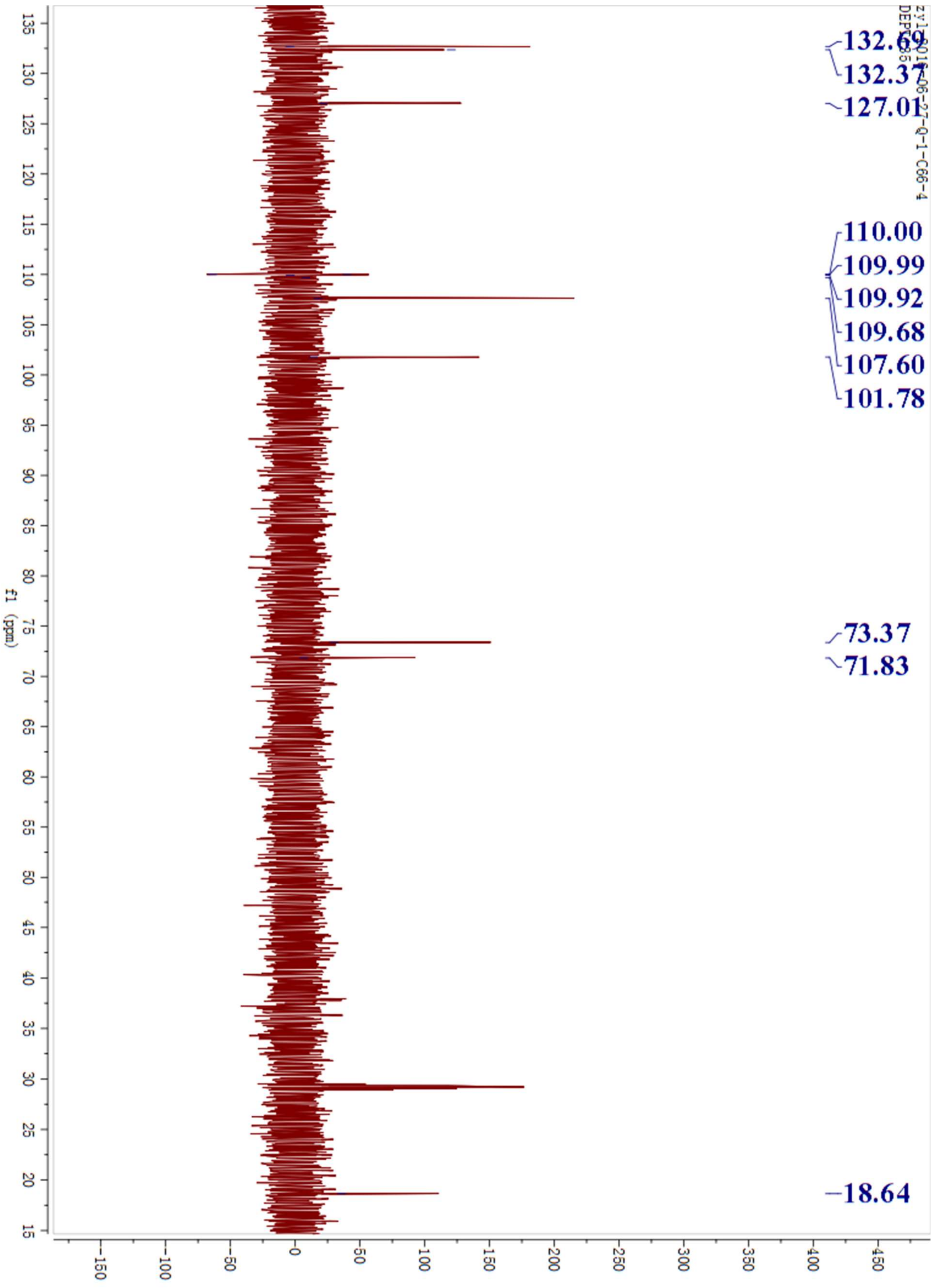

Figure S3. DEPT 135 spectrum of compound **2** in Acetone-d_6.

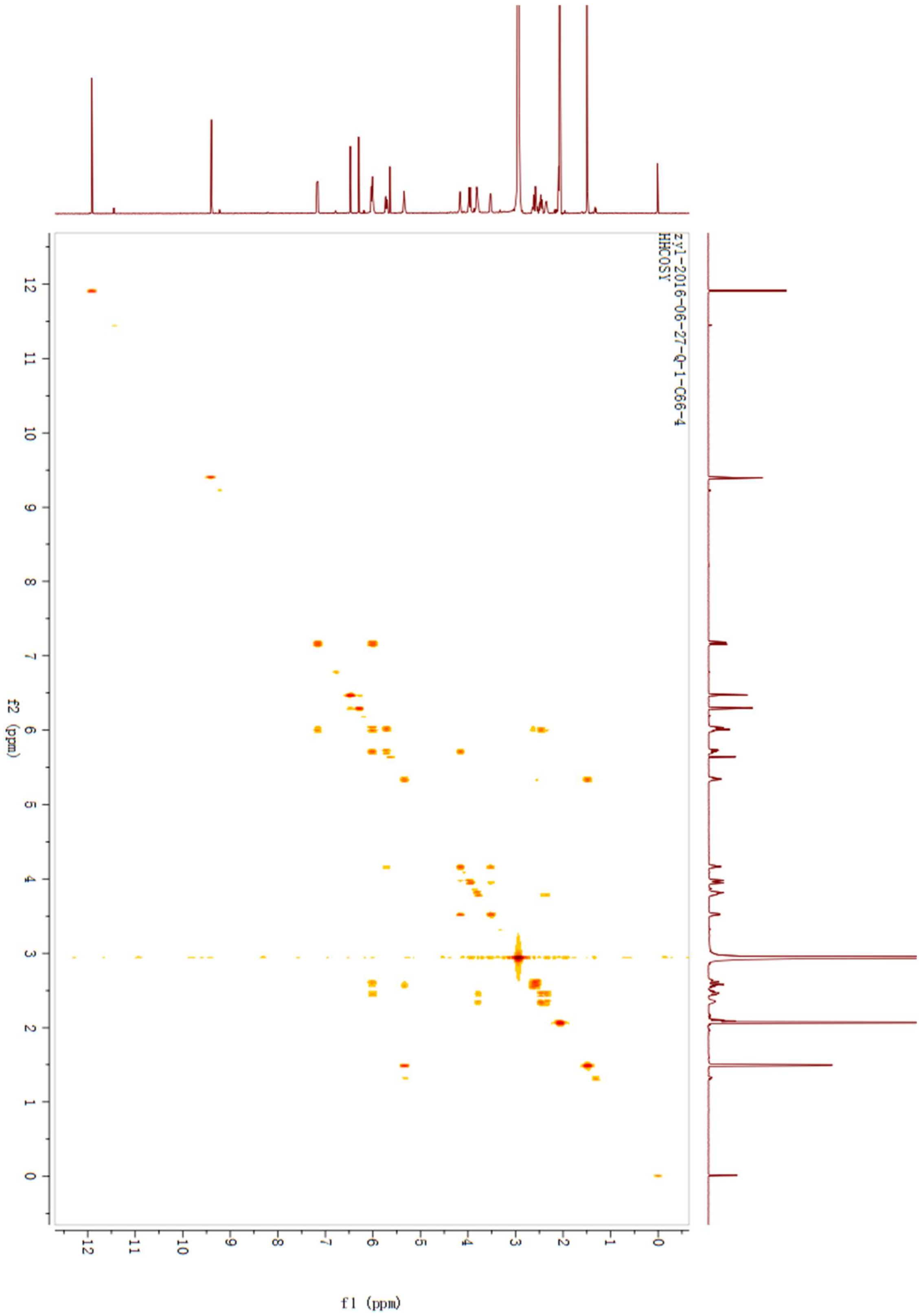

Figure S4. ^{1}H-^{1}H COSY spectrum of compound **2** in Acetone-d_6.

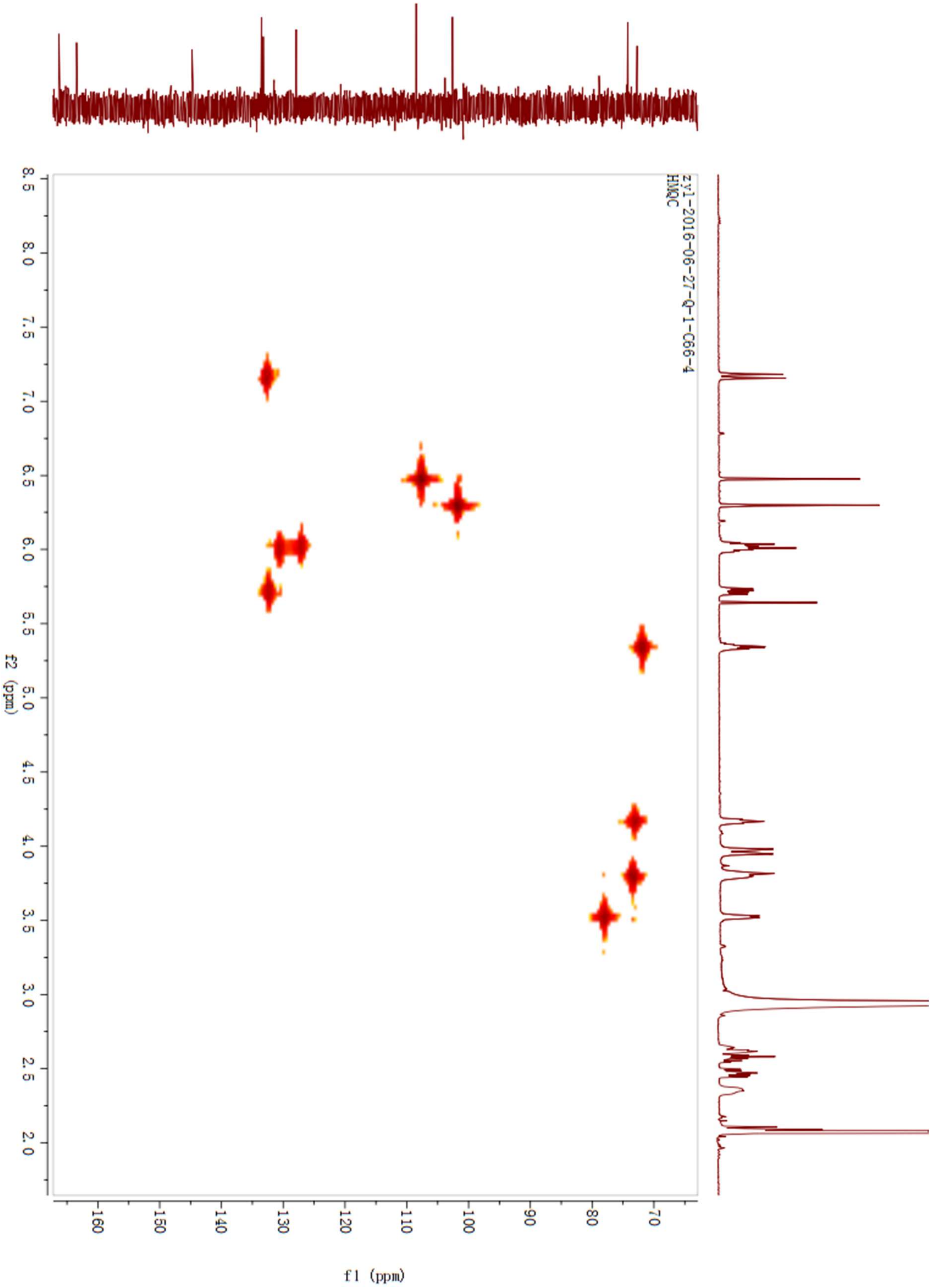

Figure S5. HMQC spectrum of compound **2** in Acetone-d_6.

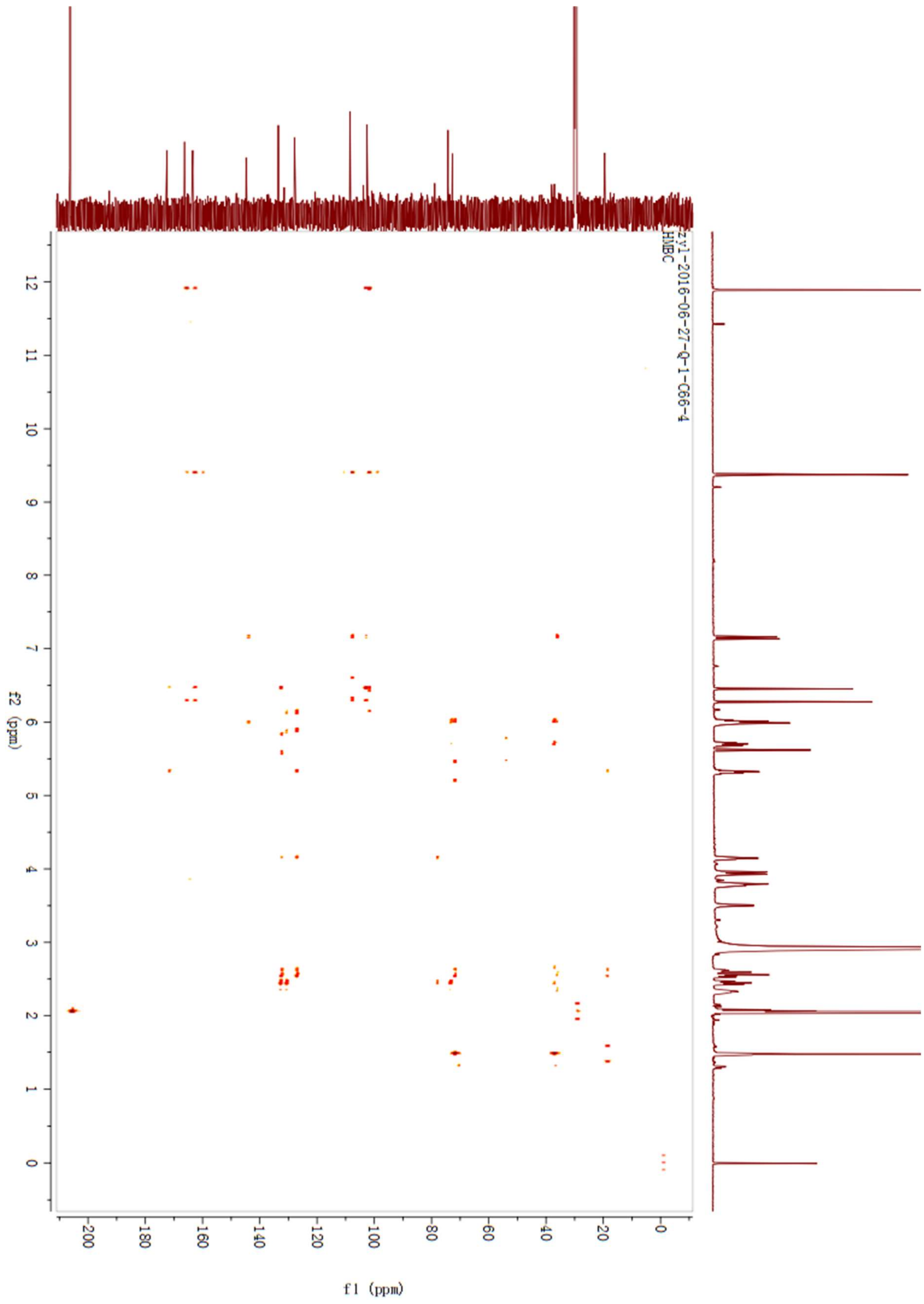

Figure S6. HMBC spectrum of compound **2** in Acetone-d_6.
